# Therapeutic Effects of Hydroalcoholic Extracts from the Ancient Apple Mela Rosa dei Monti Sibillini in Transient Global Ischemia in Rats

**DOI:** 10.3390/ph14111106

**Published:** 2021-10-29

**Authors:** Hasan Yousefi-Manesh, Ahmad Reza Dehpour, Seyed Mohammad Nabavi, Malihe Khayatkashani, Mohammad Hossein Asgardoon, Mohamad Hasan Derakhshan, Sahand Adib Moradi, Mohammad Sheibani, Seyed Mohammad Tavangar, Samira Shirooie, Joice Guileine Nkuimi Wandjou, Giovanni Caprioli, Stefania Sut, Stefano Dall’Acqua, Filippo Maggi

**Affiliations:** 1 Experimental Medicine Research Center, Tehran University of Medical Sciences, Tehran 1417613151, Iran; hasanyousefimanesh@gmail.com (H.Y.-M.); dehpour@yahoo.com (A.R.D.); Mh_asgardoon@yahoo.com (M.H.A.); derakhshanhasan@yahoo.com (M.H.D.); sahand.adibmoradi@gmail.com (S.A.M.); 2 Department of Pharmacology, School of Medicine, Tehran University of Medical Sciences, Tehran 1417613151, Iran; 3 Applied Biotechnology Research Center, Baqiyatallah University of Medical Sciences, Tehran 1435916471, Iran; nabavi208@gmail.com; 4 Department of Anesthesiology, Imam Khomeini Hospital, Tehran University of Medical Sciences, Tehran 1417613151, Iran; malihekhayatkashani@gmail.com; 5 Iranian Student Society for Immunodeficiencies, Students’ Scientific Research Center, Tehran University of Medical Sciences, Tehran 1417613151, Iran; 6 Department of Pharmacology, School of Medicine, Iran University of Medical Sciences, Tehran 1449614535, Iran; mohammad.sheibani89@gmail.com; 7 Department of Pathology, Dr. Shariati Hospital, Tehran University of Medical Sciences, Tehran 1417613151, Iran; tavangar@ams.ac.ir; 8 Chronic Diseases Research Center, Endocrinology and Metabolism Population Sciences Institute, Tehran University of Medical Sciences, Tehran 1417613151, Iran; 9 Pharmaceutical Sciences Research Center, Health Institute, Kermanshah University of Medical Sciences, Kermanshah 6715847141, Iran; 10 School of Pharmacy, University of Camerino, 62032 Camerino, Italy; joice.nkuimiwandjou@unicam.it (J.G.N.W.); giovanni.caprioli@unicam.it (G.C.); 11 Department of Pharmaceutical and Pharmacological Sciences, Natural Product Laboratory, University of Padova, Padova 35122, Italy; stefania.sut@unipd.it (S.S.); stefano.dallacqua@unipd.it (S.D.)

**Keywords:** Mela Rosa dei Monti Sibillini, apple extract, ischemic stroke, inflammation, anti-oxidative stress, caspase-3

## Abstract

The Mela Rosa dei Monti Sibillini is an ancient apple variety cultivated by Romans in the foothills of the Sibillini Mountains, central Italy, showing potential as a source of nutraceuticals. The purpose of this study was to evaluate the protective effects of the hydroalcoholic extracts from the peel (APE) and pulp (APP) of this fruit in an animal model of transient global ischemia. Chemical constituents were analyzed by liquid chromatography–mass spectrometry (LC-DAD-MS^n^) indicating several polyphenols such as B-type procyanidins, quercetin derivatives and hydroxycinnamic acids as the main bioactive components. Acute pre-treatment of extracts (30 mg/kg, i.p.) significantly decreased the brain levels of the pro-inflammatory cytokines IL-1β (*p* < 0.01) and TNF-α (*p* < 0.001 and *p* < 0.01 for APE and APP, respectively), the expression of caspase-3 (*p* < 0.01, For APE) and MDA (*p* < 0.05), a lipid peroxidation biomarker in rats. Both extracts restricted the pathological changes of the brain induced by ischemic stroke in hematoxylin and eosin assay. Moreover, they improved the scores of behavioral tests in grid-walking and modified neurological severity scores (mNSS) tests. In conclusion, these results proved this ancient Italian apple is a source of nutraceuticals able to protect/prevent damage from brain ischemia.

## 1. Introduction

One of the most common reasons for death and debility in the world is the stroke [1]. There are two types of stroke, ischemic and hemorrhagic [2]. Cerebral blood flow blockade and rupture of blood vessels occur in ischemic and hemorrhagic strokes, respectively. About 87% of strokes belong to the first type [3]. Many pathological conditions are associated with strokes such as atrial fibrillation, high blood pressure, aneurysms, diabetes mellitus, abnormal lipid profile, obesity and alcohol abuse [4,5,6,7,8]. In addition, several neurological responses occur due to stroke and hypoxia including release of pro-inflammatory cytokines, infiltration of phagocytes, glutamate overactivity, reactive oxygen species (ROS) and reactive nitrogen species (RNS) generation resulting in neural death [9].

Caspases are important enzymes in apoptosis [10], and notably caspase-3 is the greatest abundant caspase expressing in neurons which is activated during stroke and triggers programmed cell death [11]. In addition to apoptosis, caspase-3 can change glial function and elevates inflammation and nerve injury [12]. Caspase-3 is involved in many physiological process such as cellular differentiation and dendritic pruning [13]. The level of caspase-3 is increased in astrocytes after a stroke and inhibition of caspase-3 shows a neuroprotective effect in ischemic stroke [14].

Many anti-inflammatory and antioxidant agents have been studied in animal models to reduce lethal events during a stroke [1,15]. Nevertheless, the clinical use of chemical agents is still a safety concern. In this regard, the application of natural products appears to be a good approach to decrease the mortality of strokes. Among fruits and vegetables regarded as sources of phytonutrients capable of preventing damage from oxidative stress and inflammation, the apple is considered one of the most important from a nutraceutical standpoint [16]. 

Notably, it has been seen that ancient apple varieties, which are mostly restricted to local areas where they were cultivated for a long time, are richer in pharmaceutically valuable constituents than the respective commercial varieties that are instead cultivated worldwide, often in areas not suitable in terms of climatic conditions [17,18]. Our group has recently focused on the Mela Rosa dei Monti Sibillini, an ancient apple variety cultivated by Romans in the foothills of the Sibillini Mountains, central Italy, between 400 and 900 m of altitude, and recognizable by its small size, its irregular shape, its shades ranging from pink to purplish red, its intense and aromatic aroma and its sour and sugary flavor [19]. This fruit has been revealed to be rich in polyphenols such as flavan-3-ols/procyanidins, dihydrochalcones, flavonols, and hydroxycinnamic acids, as well as triterpene acids including annurcoic and ursolic acids, being valuable as source of nutraceuticals useful to manage conditions related to inflammation and oxidative stress [16]. As a matter of fact, in several studies apple peel extract (APE) and apple pulp extract (APP) have shown anti-inflammatory and anti-oxidative stress effects in animal models of renal ischemia/reperfusion injury, carbon tetrachloride (CCl4)-induced hepatotoxicity, diabetic pancreas model and colitis via down regulation of NF-kB, pro-inflammatory cytokines, increasing the antioxidant enzyme activity including superoxide dismutase (SOD), catalase (CAT) and reducing myeloperoxidase (MPO) activity [1,17,18,19]. Due to the roles of oxidative stress and inflammation in ischemic stroke and the anti-oxidative stress and anti-inflammation properties of APP and APE from the Mela Rosa dei Monti Sibillini, here we aimed to assess its protective effect against acute ischemic stroke.

One of the most common experimental models of ischemic stroke is bilateral common carotid artery occlusion (BCCAO) [20]. In the current research, we considered the beneficial effects of APP and APE on a BCCAO animal model of ischemic stroke. Protective effects of APP and APE were evaluated by behavioral assays (grid-walking and modified neurological severity scores (mNSS) tests). In addition, the brain level of caspase-3, an apoptotic marker in neurons, was measured by immunostaining. The brain levels of TNF-α, IL-1β as inflammatory factors and MDA (malondialdehyde), a lipid peroxidation marker, as an oxidative stress factor, were measured. The chemical composition of APP and APE was studied by high-performance liquid chromatography-diode array detection-mass spectrometry (HPLC-DAD-MS) analysis.

## 2. Results

### 2.1. Chemical Constituents of Apple Peel Extract (APE) and Apple Pulp Extract (APP)

**Figure 1:**
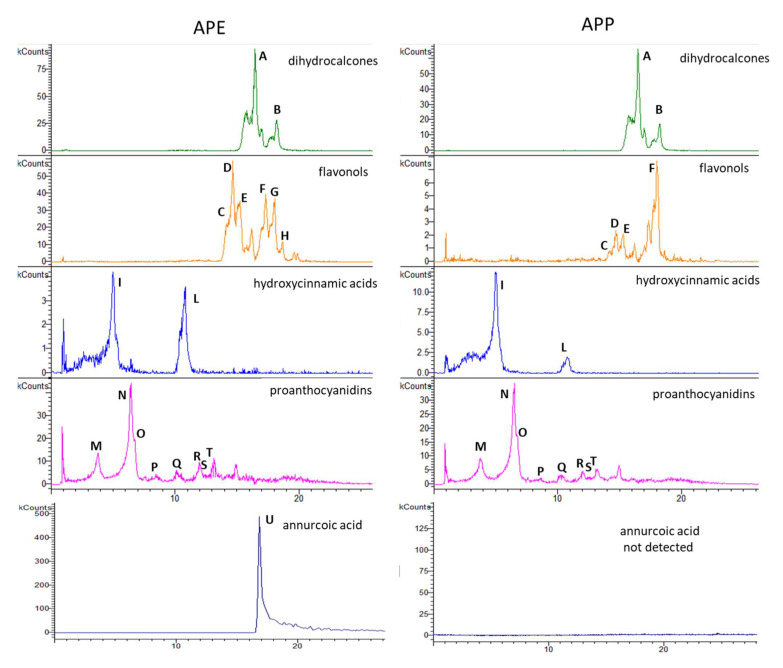
Exemplificative chromatograms of apple peel extract (APE) and apple pulp extract (APP) by LC-ESI-MSn. Dihydrochalcone and flavonol derivatives: 567 *m*/*z* phloretin-2-O-xylo-glucoside (A); 435 *m*/*z* phloretin-2-O-glucoside (B); 463 *m*/*z* quercetin-3-O-galactoside (C); 609 *m*/*z* rutin (D); 433 m/z quercetin-3-O-xyloside(E); 433 *m*/*z* quercetin-3-O-arabinoside (F); 447 *m*/*z* quercetin-3-O-rhamnoside (G); 477 *m*/*z* rhamnetin-3-O-glucoside (H); hydroxycinnamic acids: 535 *m*/*z* chlorogenic acid isomer 1 (I); 353.5 *m*/*z* chlorogenic acid isomer 2 (L). Proanthocyanidin derivatives: 289 *m*/*z* catechin (M); 577 *m*/*z* procyanidin B isomer 1 (N); 577 *m*/*z* procyanidin dimer B isomer 2 (O); 1441 *m*/*z* procyanidin derivative (P); 865 *m*/*z* procyanidin trimer B (Q); 1441 *m*/*z* procyanidin pentamer B (R); 1153 *m*/*z* procyanidin tetramer B (S); 1729 *m*/*z* procyanidin derivative (T); 485 *m*/*z* annurcoic acid (U) by APCI ion sources. In the reported chromatograms, x axis was time (min) and y axis was kCounts.

The presence of bioactive constituents in apple fruit extracts can be related to the observed effects in the bioassays. In a previous paper APE and APP secondary metabolites composition were investigated by LC-DAD-MS^n^ resulting in a complex pattern of phytoconstituents [20]. Dihydrochalcones, hydroxycinnamic acids, quercetin derivatives and B type procyanidins were identified as the most abundant polyphenols. The chromatographic fingerprint of the extracts is depicted in Figure 1. 

**Figure 2:**
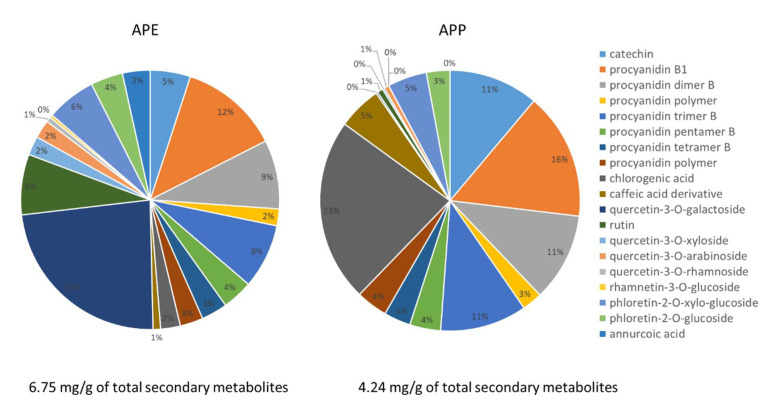
Graphical representation of percentage of each secondary metabolite identified in APE and APP.

To assess a possible role of these constituents, we obtained a fingerprint of the extract and the overall content of phenolics and triterpene acids measured in the samples was 6.75 mg/g in APE and 4.24 mg/g in APP. The most abundant compounds in APE and APP were procyanidin B dimer (isomer 1), 12 and 16%, respectively, quercetin-3-O-galactoside (23% in APE) and chlorogenic acid (23% in APP). Quercetin-3-O-rhamnoside (9%), rhamnetin-3-O-glucoside (2%) and annurcoic acid (3%) were detected in APE, but not in APP (Figure 2). 

### 2.2. APP and APE Improved the Behavioral Tests after the Brain Transient Global Ischemia

**Figure 3:**
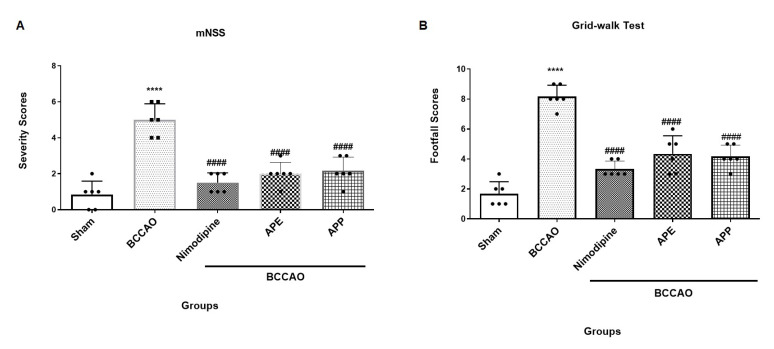
(**A**) Modified neurological severity scores (mNSS). Results are mean ± SD. (*n* = 6). **** *p* < 0.0001 vs. the sham group, ^####^ *p* < 0.0001 vs. the bilateral common carotid artery occlusion (BCCAO) group. (**B**) Grid-walking test. Results are mean ± SD. Repeated measures one-way analysis of variance with Bonferroni post-hoc was used. **** *p* < 0.0001 vs. the sham group, ^####^ *p* < 0.0001 vs. the BCCAO group.

mNSS and grid walking tests were performed to assess the neurological function after ischemic stroke (Figure 3A,B). The BCCAO group significantly showed a more severe score in mNSS test (F (4, 25) = 0.3676, *p* < 0.0001) and a higher footfall score in grid walking test than the sham group (F (4, 25) = 1.438, *p* < 0.0001). However, pre-treatment with APE, APP and nimodipine displayed protective effects in neurological functions and significantly improved the scores in both mNSS and grid walking tests when compared with the BCCAO group (*p* < 0.0001, for all).

### 2.3. APP and APE Decreased Brain Tissue Injury in Hematoxylin and Eosin (H and E) Staining 

**Figure 4:**
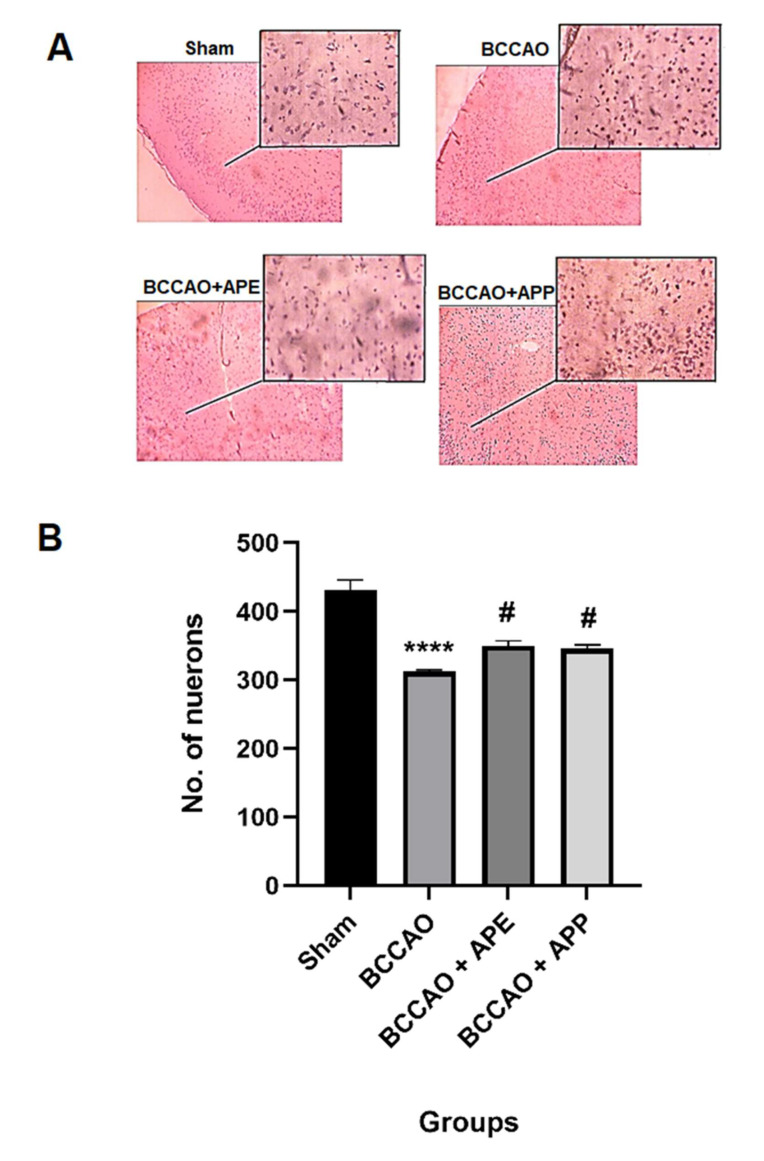
APE and APP pre-treatment reduced the brain motor cortex damage 24 h after ischemia/reperfusion in animals and improved the number of survival neurons compared to the BCCAO group (**A**,**B**). Micrographs (10× and 40×) of hematoxylin and eosin (H and E) stained brain tissue. Repeated measures one-way analysis of variance with Bonferroni post-hoc was used. **** *p* < 0.0001 vs. the sham group; ^#^ *p* < 0.05 vs. the BCCAO group.

As indicated in Figure 4, the H and E assay of brain motor cortex showed that the rat brain from the sham group had normal histology with preserved neurological cells. In the BCCAO group, partial loss of neurological cells with cavity formation was observed. Pre-treatment with APP and APE showed relatively preserved neurological cells with normal histology.

### 2.4. APP and APE Reduced the Pro-Inflammatory Cytokines and Oxidative Stress in Brain after Ischemic Stroke

**Figure 5:**
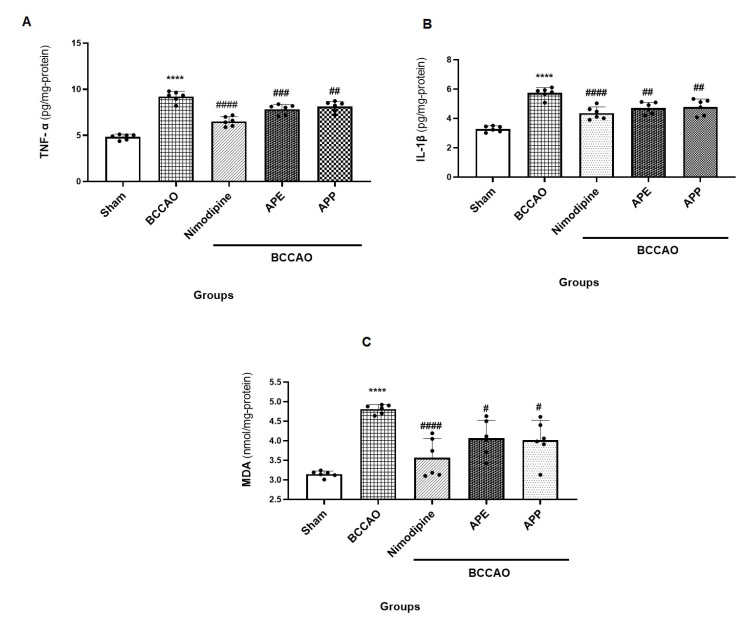
(**A**) The brain levels of TNF-α in experimental groups 60 min after ischemia/reperfusion. Results are mean ± SD. (*n* = 6). **** *p* < 0.0001 vs. the sham group, ^####^ *p* < 0.0001, ^###^ *p* <0.001 and ^##^ *p* < 0.01 vs. the BCCAO group. (**B**) The brain levels of IL-1β in experimental groups 60 min after ischemia/reperfusion. Results are mean ± SD. **** *p* < 0.0001 vs. the sham group, ^####^ *p* < 0.0001 and ^##^ *p* < 0.01 vs. the BCCAO group. (**C**) The brain levels of malondialdehyde (MDA) in experimental groups 60 min after ischemia/reperfusion. Results are mean ± SD. Repeated measures one-way analysis of variance with Bonferroni post-hoc was used. **** *p* < 0.0001 vs. the sham group, ^####^ *p* < 0.0001 and ^#^ *p* < 0.05 vs. the BCCAO group.

The brain motor cortex levels of MDA and pro-inflammatory cytokines including TNF-α and IL-1β were measured by ELISA. As shown in Figure 5A–C, brain levels of TNF-α, IL-1β and MDA in the BCCAO group were significantly higher when compared with the sham group (F (4, 25) = 0.3239), (F (4, 25) = 1.344), (F (4, 25) = 3.711) (*p* < 0.0001, for all). Our data showed that pre-treatment with nimodipine, APP and APE reduced significantly the levels of TNF-α (*p* < 0.0001, *p* = 0.0098 and *p* = 0.0008, respectively), IL-1β (*p* < 0.0001, *p* = 0.0026 and *p* = 0.0012) and MDA (*p* < 0.0001, *p* = 0.0108 and *p* = 0.0184, respectively) when compared with the BCCAO group.

### 2.5. APP and APE Reduced Caspase 3 Expression in the Brain after Transient Global Ischemia

**Figure 6:**
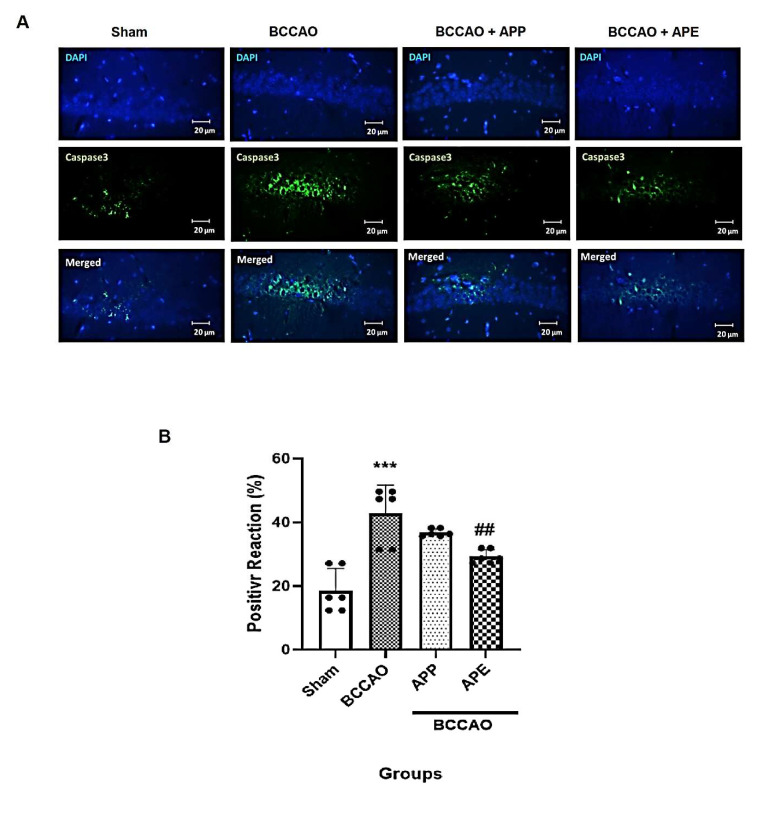
APE reduced the number of caspase-3 expressing cells in hippocampi. (*n* = 6). (**A**) Microphotographs show the co-localization of caspase-3 (green)-positive cells with DAPI (blue) in hippocampi of animals. (**B**) Semi-quantitative assay of active caspase-3. Results are mean ± SD. Repeated measures one-way analysis of variance with Bonferroni post-hoc was used. *** *p* < 0.001 vs. the sham group, ^##^ *p* < 0.01 vs. the BCCAO group.

Cerebral ischemia activates apoptotic pathways leading to neuronal cell death [21]. To evaluate the effects of APP and APE treatment on apoptosis induced by ischemia, caspase-3 as an apoptotic marker was measured by fluorescence immunostaining (Figure 6A,B). Caspase-3 expression was significantly elevated in the hippocampus of the BCCAO group when compared with the sham group (F (3, 20) = 1.826, *p* < 0.0001). However, pre-treatment with APE significantly reduced the expression of caspase-3 compared with the BCCAO group (*p* = 0.0029). On the other hand, APP reduced insignificantly the caspase-3 expression when compared with the BCCAO group (*p* = 0.2839).

## 3. Discussion

This study showed that pre-treatment with APE and APP had a protective effect on brain tissue by reducing pro-inflammatory cytokines and oxidative stress. Ischemic reperfusion of the brain leads to neuronal apoptosis, activation, and infiltration of inflammatory cells. Reperfusion of the brain results in brain damage due to the ROS, RNS generation and apoptosis [22,23]. Caspase-3 is produced as an inactive proenzyme that could be activated by extrinsic and intrinsic programmed cell death pathways [24]. It has been shown that the caspase-3 level is high during acute pathophysiological conditions such as ischemic stroke leading to neuronal apoptosis, and treatment with caspase-3 inhibitors decreased the infarct area following stroke induction [25]. Le et al. have reported that caspase 3-deficient mice were resistant to the pathological effects of stroke and had smaller infarct area than wild-type mice 48 h after stroke. These authors have also shown that cortical neurons cultured from caspase-3(−/−) mice were more resistant than normal neurons against oxygen-glucose deficiency for 2 h [11]. Previous studies have demonstrated that lipid peroxidation and an increase of the brain level of MDA occurred after ischemic-reperfusion of cerebral brain vessel, and proved direct correlation between the MDA brain level and the infarct volume [26,27]. The other main pathogenic factor of ischemic stroke is inflammation. Both the adaptive and innate immune responses are activated after ischemic stroke and microglia brain immune cells activation results in damage-associated molecular patterns (DAMPs) [28]. Activated microglia secrete pro-inflammatory cytokines (TNF-α, IL-1β and IL-6), ROS and RNS resulting in neuronal cytokine-related apoptosis that stimulates the recruitment of T cell lymphocytes and results in establishment of an inflammatory system around the neuronal damage [29]. Due to ROS and RNS generation in strokes, polyunsaturated fatty acids peroxidation in membrane cells occurs resulting in neuronal structural and functional impairment. The most toxic products of lipid peroxidation are MDA and thiobarbituric acid-substrates which increase significantly during ischemic strokes [30,31].

In our study, the brain level of caspase-3 in the BCCAO group was markedly higher than the sham group, and this was reversed by pre-treatment with APE. The study also demonstrated that pretreatment with APP and APE considerably decreases the levels of TNF-α, IL-1β and MDA which are pro-inflammatory and oxidative stress indicators. It has been demonstrated that natural polyphenolic compounds have anti-apoptotic effects and reduced neuronal cell death after induction of cerebral ischemic stroke via downregulation of poly ADP-ribose polymerase (PARP) protein and caspase-3 expression, reduction of MDA level and ROS generation, and upregulated the anti-oxidative stress genes such as Nrf2, HO-1, and NQO1 [32,33]. Apple is enriched by neuroprotective natural polyphenolic compounds such as flavonols, anthocyanins, phenolic acids and flavan-3-ols [34]. Paul and colleagues have shown that Northern Spy apple peel extract has neuroprotective and anti-inflammatory effects in an in vivo model of hypoxic-ischemic brain damage via suppression of the IL-1β, TNF-α and IL-6 expression, elevated levels of anti-apoptotic factors including Bcl-2 and XIAP in the hippocampus and striatum of mice, and improved motor function scores [35]. Several researches indicated that extracts from Mela Rosa dei Monti Sibillini have potential anti-inflammatory and anti-oxidative stress properties in CCl4-induced hepatotoxicity and renal ischemia/reperfusion injury in rats [16,17]. Considering the phytochemical composition, APE presents higher levels of flavonoids when compared to APP. The most abundant phenolics in APE were quercetin-3-galactoside (23%) and procyanidin dimer isomer 1 (12%). A recent review reported significant role of quercetin in attenuation of ischemic/reperfusion injury, suggesting that this compound can improve cell membrane integrity decreasing lipid peroxidation. Furthermore, quercetin inhibited apoptosis through downregulation of Bax, and caspases, and upregulation of Bcl-2. Flavonol glycosides, like quercetin and its derivatives, which are abundant in these apple extracts, showed neuroprotective effects by reducing the brain levels of nitric oxide, MDA and increasing SOD activity [36]. Finally, authors reported that quercetin can modulate autophagy decreasing ischemic reperfusion injury by different mode of actions [36]. Considering procyanidins involvement, grape seed procyanidin extract was reported to be active in the reduction of ischemic reperfusion injury in mice CNS tissues [36]. It has been reported that these compounds have the ability to inhibit caspase-3 and improve neuronal cell survival after an intoxication [36]. It is well known that procyanidins display anti-inflammatory effects by reducing the levels of indicators such as TNF-α, Il-1β, COX-2, MDA, NO and enhancing SOD concentrations [36]. They have demonstrated as well anti-oxidative activities [36]. A study of the neuroprotective activity of triterpenic acid and ursolic acid has demonstrated that this compound reduces infart size and lower lipid peroxidation [36]. It also suppresses the level of expression of TLR4 and NF-*κ*B after the injury, displaying, thus, anti-inflammatory capacities [36]. A further constituent that may play a role in the bioactivity of the extract may be annurcoic acid that account in APE at 3% of total secondary metabolites. Indeed, a previous paper revealed that other triterpene acids are able to modulate ischemic reperfusion damages as maslinic acid, madecassoside, and betulinic acid [36]. Thus, further investigations are needed to evaluate the role of apple triterpene acids for this specific bioactivity.

In our study, both APE and APP reduced the brain levels of IL-1β, TNF-α and MDA and improved motor performance scores and histological changes. 

## 4. Materials and Methods

### 4.1. Apple Sampling

Apple sampling was undertaken as previously described by Yousefi-Manesh et al. [16,17]. Apple trees were cultivated in the orchards of farmer Marini, situated in Montefalcone Appennino (42°59′17″ N; 13°27′32″ E, 700 m a.s.l.), Fermo district, Marche region, central Italy. The harvest was carried out in the apple season, in November 2017. The samples storage was at room temperature (around 20 °C) until extraction.

### 4.2. Hydroalcoholic Extracts Preparation

The apple peel extract (APE) and apple pulp extract (APP) were prepared as previously described by Yousefi-Manesh et al. [17]. Briefly, pulp and peel were separated using a knife and dried in a Biosec De Luxe B12 dryer (Albrigi luigi, Verona, Italy) at 40 °C for 18 h until a constant weight. Afterwards, apple-dried materials were reduced into a powder (particle size, 1 mm) by IKA-WERK MFC DCFH 48 (Staufen, Germany). Powders were stored in 50 mL Falcon tubes at room temperature until extraction. Ten g of apple peel and pulp powder were extracted by sonication using a mixture of methanol-water solution (200 mL, 1:1 *v*/*v*) at 45 °C for 60 min. APE and APP residuals were resuspended and sonicated in 50 mL of the same extractant solution at 45 °C for a further 30 min. The solutions obtained were pulled and finally dried under vacuum at 40 °C by rotavapor. The yields obtained were 64% and 54% (*w*/*w*) for APP and APE, respectively.

### 4.3. High-Performance Liquid Chromatography-Diode Array Detection-Mass Spectrometry (HPLC-DAD-MS^n^) Analysis

Composition of phenolic derivatives was obtained by HPLC-DAD-MS^n^, as reported previously using an Agilent 1260 chromatograph (Santa Clara, CA, USA) with a diode array (DAD) and Varian MS-500 ion trap mass spectrometer. A “T” connector divided the eluate in equal amounts to DAD and MS. Agilent Eclipse XDB C-18 (3.0 × 150 mm) 3.5 µm was used as a stationary phase [17]. Mobile phases were acetonitrile (A) and water 0.1% formic acid (B) and flow rate was 0.5 mL/min. Gradient elution was used as reported by Yousefi-Manesh et al. [17]. Rutin, chlorogenic acid, phloridzin and catechin (Sigma-Aldrich, Milan, Italy) were used as reference compounds for flavonoid, hydroxycinnamic acid derivatives, chalcones and proanthocyanidin derivatives, respectively. Phenolic compounds were quantified at 280, 330, and 350 nm, on the base of UV spectra (range of 200–650 nm). Calibration curves were as follows: rutin y = 27.788x + 330.7 (r^2^ = 0.9981); chlorogenic acid y = 47.359x + 439.99 (r^2^ = 0.9951); catechin y = 20.525x + 3.2962 (r^2^ = 0.999); phloridzin y = 87.029x − 1.832 (r^2^ = 0.999). MS spectra were recorded in negative ion mode in 50–2000 Da range, using an ESI ion source. Fragmentation of the main ionic species were obtained by the turbo data-dependent scanning (TDDS) function. 

A previously published method was used for quantification of triterpene acids. Briefly, an Agilent Eclipse XDB C-18 (3.0 × 150 mm) 3.5 μm was used as stationary phase [7]. Methanol (A) and H_2_O 0.05% formic acid (B) were the mobile phases. The analysis revealed the presence of annurcoic acid; the latter was quantified using the calibration curve of the purified compound considering the ion species at *m*/*z* 485 in the range 5–50 µg/mL, calibration curve was as follow, y = 12,549x + 136,925; r^2^ = 0.9962 [17].

### 4.4. Animals

Sixty adult male Wistar rats weighing 220–250 g, 8 weeks old, were obtained from the Animal Center in Tehran University of Medical Sciences, Iran. Standard conditions such as 12 h day/night cycle, 23–24 °C and freely admission to food and water were established for animals. All investigational trials were accepted by the Animal Care and Use Committee of Tehran University of Medical Sciences (no.: PT20180715). The behavioral studies were done from 9:00 a.m. to 14:00 p.m. 

#### 4.4.1. Induction of Ischemic Stroke 

The BCCAO stroke model was performed as formerly reported by Li et al. [37]. In brief, rats were anesthetized with ketamine (45 mg/kg, i.p. (intraperitoneal)). A midline cut in the neck was made to expose the bilateral common carotid arteries; after separation of the peripheral tissues, both arteries were obstructed by a clamp for 30 min. Then, reperfusion was undertaken by removing the clamp for 1 h. Half of the animals in each group were decapitated and the brain tissues were fully separated for acute molecular assays, and behavioral tests were performed on the other half of the animals 24 h later.

#### 4.4.2. Animal Groups

Rats were divided into the following five groups (12 rats each):

1. Sham group: the animals were only anesthetized, a midline cut in the neck was made without BCCAO and received normal saline i.p.
2. BCCAO group: the stroke model was induced as described above; animals received normal saline i.p.
3. Nimodipine group (as positive group): 30 min before BCCAO, the animals received nimodipine 10 mg/kg (i.p.), then BCCAO was induced [22].
4. BCCAO + APP: 30 min before BCCAO, the animals received APP extract 30 mg/kg (i.p.), then BCCAO was induced [17].
5. BCCAO + APE: 30 min before BCCAO, the animals received APE extract 30 mg/kg (i.p.), then, BCCAO was induced [17].

All of the doses for this experiment were chosen based on previous studies [17,22]. Half of the animals in each group were sacrificed 1 h after reperfusion to analyze the hippocampus levels of caspase-3 and brain cortex molecular assay. The second half of the remaining animals were sacrificed 24 h after reperfusion for H and E assay and behavioral tests. 

### 4.5. Behavioral Tests

To evaluate behavioral disability and the protective effects of APP and APE pre-treatment, a grid walking test and modified neurological severity score (mNSS) were done 24 h after BCCAO induction. For the grid walking test, or the foot fault task, the animals were placed above a gridiron floor (2025 cm^2^) with holes (1 cm) across and height of 2.5 cm; the animals were permitted to walk freely on the gridiron floor for one min. Once the animals’ paws slipped and touched the floor, the score was 2 and each time their paws slipped without touching the floor the score was 1. The more severe the neurological injury, the higher score was recorded [38]. The mNSS test was composed of sensory, motor, and balance tests with a total score of 14. The animal scored one for each inability. The higher score shows the greater severity of neurological damage. Scores of 1~4, 5~9 and 10~14 suggest mild, moderate and severe defects, respectively [39].

### 4.6. Caspase-3 Immunostaining

The hippocampus of half sacrificed rats in each group were fixed in 10% formalin after 1 h reperfusion for 72 h to analyze the changes of caspase-3 expression via immunostaining. Next, the tissues were inserted in paraffin, and 5 μm slices were prepared by a microtome. The samples were attached onto slides that coated with saline and after dewaxing and rehydrating in graded series of ethanol, washed with distilled water. The samples were incubated 2 h at room temperature and 24 h at 2–8 °C with 10% normal donkey serum and caspase-3 antibody (1:100 diluted with PBS) (orb-213644), respectively. After washing four times with PBS, Goat Anti-Rabbit IgG(H+L) antibody (FITC) (orb688925) (1:150 diluted with PBS) as the second antibody was added and the samples were incubated in a dark room (37 °C, 90 min). Then, they were washed three times with DAPI (Sigma-D9542) and for 20 min with PBS. Finally, glycerol/PBS solution was spilled on the samples and they were evaluated with a fluorescence microscope (Olympus), and ImageJ software was used to calculate the expression of caspase-3. The percentage of staining area indicated the expression of caspase-3 [9].

### 4.7. Histological Assay

The brain motor cortex of half of each group of sacrificed rats was fixed in 10% formalin for 72 h for hematoxylin and eosin (H and E) staining. The tissues were inserted in paraffin, and 5 μm slices were prepared by a microtome, and to evaluate the pathological injury H and E dye was used for staining [40]. Image J software was used to quantify the images and count the mean number of neurons.

### 4.8. Molecular Analysis

The brain levels of TNF-α, IL-1β and MDA were measured by an R&D systems ELISA kit after 1 h reperfusion. The whole brain of animal was hemoginated (10%, *w*/*v* in phosphate buffer) and centrifuged at 12,000 rpm and 4 °C for 20 min. Then, the pro-inflammatory cytokines TNF-α and IL-1β levels were measured by enzyme immunoassay (Karmania Pars Gene, Tehran, Iran). The brain MDA level was measured as the lipid peroxidation marker with a MDA assay kit (Sigma, Darmstadt, Germany) [17].

### 4.9. Statistical Analysis

Statistical analysis was undertaken with GraphPad Prism 9.0 software. The data are expressed as mean ± standard deviation (SD). The one-way ANOVA followed by post hoc Bonferroni test was used to indicate the differences between groups. *p* < 0.05 was set as the statistical significance.

## 5. Conclusions

Consumption of ancient apples like the Mela Rosa dei Monti Sibillini should be promoted in rural areas and their cultivation improved as they represent an important part of a diet useful to prevent cardiovascular and neurological diseases. Furthermore, this old apple and its respective by-products could be utilized on an industrial level as source of nutraceuticals to be used as adjuvant with conventional therapies in pathological conditions related to inflammation and oxidative stress. Our results showed that the neuroprotective effects of extracts of this ancient apple variety from central Italy are generally related to their anti-inflammatory and anti-oxidative stress properties ascribable to their major constituents such as flavonol glycosides and procyanidins. In addition, these extracts are capable of reducing the apoptotic marker, caspase-3, improving motor functional test scores. Further investigations are needed on the most abundant secondary metabolites such as quercetin-3-O-galactoside, procyanidin dimers, and on triterpene annurcoic acid to evaluate their protective role in ischemia reperfusion damage. Thus, our findings pave the way for the improvement of cultivation systems of this old apple and its exploitation for nutraceutical purposes.

## Data Availability

Not applicable.

## References

[1] Di Lorenzo A., Sobolev A.P., Nabavi S.F., Sureda A., Moghaddam A.H., Khanjani S., Di Giovanni C., Xiao J., Shirooie S., Tsetegho Sokeng A.J. (2019). Antidepressive effects of a chemically characterized maqui berry extract (Aristotelia chilensis (molina) stuntz) in a mouse model of Post-stroke depression. Food Chem. Toxicol..

[2] Virani S.S., Alonso A., Aparicio H.J., Benjamin E.J., Bittencourt M.S., Callaway C.W., Carson A.P., Chamberlain A.M., Cheng S., Delling F.N. (2021). Heart Disease and Stroke Statistics-2021 Update: A Report From the American Heart Association. Circulation.

[3] Dojo Soeandy C., Salmasi F., Latif M., Elia A.J., Suo N.J., Henderson J.T. (2019). Endothelin-1-mediated cerebral ischemia in mice: Early cellular events and the role of caspase-3. Apoptosis.

[4] Kamel H., Okin P.M., Elkind M.S., Iadecola C. (2016). Atrial fibrillation and mechanisms of stroke: Time for a new model. Stroke.

[5] Willmot M., Leonardi-Bee J., Bath P.M. (2004). High blood pressure in acute stroke and subsequent outcome: A systematic review. Hypertension.

[6] Patlolla S.H., Lee H.-C., Noseworthy P.A., Wysokinski W.E., Hodge D.O., Greene E.L., Gersh B.J., Melduni R.M. (2020). Impact of diabetes mellitus on stroke and survival in patients with atrial fibrillation. Am. J. Cardiol..

[7] Chaudhary D., Khan A., Gupta M., Hu Y., Li J., Abedi V., Zand R. (2021). Obesity and mortality after the first ischemic stroke: Is obesity paradox real?. PLoS ONE.

[8] Jackova J., Sedova P., Brown R.D., Zvolsky M., Volna M., Baluchova J., Belaskova S., Bednarik J., Mikulik R. (2020). Risk Factors in Ischemic Stroke Subtypes: A Community-Based Study in Brno, Czech Republic. J. Stroke Cerebrovasc. Dis..

[9] Yousefi-Manesh H., Rashidian A., Hemmati S., Shirooie S., Sadeghi M.A., Zarei N., Dehpour A.R. (2019). Therapeutic effects of modafinil in ischemic stroke; possible role of NF-κB downregulation. Immunopharmacol. Immunotoxicol..

[10] Zheng T.S., Schlosser S.F., Dao T., Hingorani R., Crispe I.N., Boyer J.L., Flavell R.A. (1998). Caspase-3 controls both cytoplasmic and nuclear events associated with Fas-mediated apoptosis in vivo. Proc. Natl. Acad. Sci. USA.

[11] Le D.A., Wu Y., Huang Z., Matsushita K., Plesnila N., Augustinack J.C., Hyman B.T., Yuan J., Kuida K., Flavell R.A. (2002). Caspase activation and neuroprotection in caspase-3- deficient mice after in vivo cerebral ischemia and in vitro oxygen glucose deprivation. Proc. Natl. Acad. Sci. USA.

[12] Akpan N., Troy C.M. (2012). Caspase Inhibitors: Prospective Therapies for Stroke. Neuroscientists.

[13] Lo E.H. (2010). Degeneration and repair in central nervous system disease. Nat. Med..

[14] Ma J., Endres M., Moskowitz M.A. (1998). Synergistic effects of caspase inhibitors and MK-801 in brain injury after transient focal cerebral ischaemia in mice. Br. J. Pharm..

[15] Nabavi S.F., Sureda A., Sanches-Silva A., Pandima Devi K., Ahmed T., Shahid M., Sobarzo-Sánchez E., Dacrema M., Daglia M., Braidy N. (2019). Novel therapeutic strategies for stroke: The role of autophagy. Crit. Rev. Clin. Lab. Sci..

[16] Yousefi-Manesh H., Dehpour A.R., Ansari-Nasab S., Hemmati S., Sadeghi M.A., Shahraki R.H., Shirooie S., Nabavi S.M., Nkuimi Wandjou J.G., Sut S. (2020). Hepatoprotective Effects of Standardized Extracts from an Ancient Italian Apple Variety (Mela Rosa dei Monti Sibillini) against Carbon Tetrachloride (CCl4)-Induced Hepatotoxicity in Rats. Molecules.

[17] Yousefi-Manesh H., Hemmati S., Shirooie S., Nabavi S.M., Bonakdar A.T., Fayaznia R., Asgardoon M.H., Dehnavi A.Z., Ghafouri M., Wandjou J.G.N. (2019). Protective effects of hydroalcoholic extracts from an ancient apple variety ‘Mela Rosa dei Monti Sibillini’against renal ischemia/reperfusion injury in rats. Food Funct..

[18] Leontowicz H., Leontowicz M., Gorinstein S., Martin-Belloso O., Trakhtenberg S. (2007). Apple peels and pulp as a source of bioactive compounds and their influence digestibility and lipid profile in normal and atherogenic rats-in English. Med. Weter..

[19] Fathy S.M., Drees E.A. (2015). Protective effects of Egyptian cloudy apple juice and apple peel extract on lipid peroxidation, antioxidant enzymes and inflammatory status in diabetic rat pancreas. BMC Complementary Altern. Med..

[20] Liu B., Gao J.-M., Li F., Gong Q.-H., Shi J.-S. (2018). Gastrodin attenuates bilateral common carotid artery occlusion-induced cognitive deficits via regulating Aβ-related proteins and reducing autophagy and apoptosis in rats. Front. Pharmacol..

[21] Yuan J. (2009). Neuroprotective strategies targeting apoptotic and necrotic cell death for stroke. Apoptosis: Int. J. Program. Cell Death.

[22] Yousefi-Manesh H., Dehpour A.R., Shirooie S., Bagheri F., Farrokhi V., Mousavi S.E., Ricciutelli M., Cappellacci L., López V., Maggi F. (2021). Isofuranodiene, a Natural Sesquiterpene Isolated from Wild Celery (*Smyrnium olusatrum* L.), Protects Rats against Acute Ischemic Stroke. Pharmaceuticals.

[23] Hussein Y.A., Al-sarraf A.M., Alfalluji W.L. (2020). Modulation of oxidative stress, inflammatory and apoptotic response by curcumin against cerebral ischemia reperfusion injury in a mouse model. Interdiscip. Neurosurg..

[24] Fan W., Dai Y., Xu H., Zhu X., Cai P., Wang L., Sun C., Hu C., Zheng P., Zhao B.Q. (2014). Caspase-3 modulates regenerative response after stroke. Stem Cells.

[25] Kerr L., McGregor A., Amet L., Asada T., Spratt C., Allsopp T., Harmar A., Shen S., Carlson G., Logan N. (2004). Mice overexpressing human caspase 3 appear phenotypically normal but exhibit increased apoptosis and larger lesion volumes in response to transient focal cerebral ischaemia. Cell Death Differ..

[26] Awooda H.A., Lutfi M.F. (2015). Oxidative/nitrosative stress in rats subjected to focal cerebral ischemia/reperfusion. Int. J. Health Sci..

[27] Zhang J.-Y., Si Y.-L., Liao J., Yan G.-T., Deng Z.-H., Xue H., Wang L.-H., Zhang K. (2012). Leptin administration alleviates ischemic brain injury in mice by reducing oxidative stress and subsequent neuronal apoptosis. J. Trauma Acute Care Surg..

[28] Chamorro A., Meisel A., Planas A.M., Urra X., Van De Beek D., Veltkamp R. (2012). The immunology of acute stroke. Nat. Rev. Neurol..

[29] Liu C.-Y., Wang X., Liu C., Zhang H.-L. (2019). Pharmacological Targeting of Microglial Activation: New Therapeutic Approach. Front. Cell. Neurosci..

[30] Zhu X., Tao W., Yu L., Jin J., Xu Y. (2020). NOX4 negatively regulates memory functions in APP/PS1 mice: Molecular and cell biology/oxidative stress. Alzheimer’s Dement..

[31] Lee W.-C., Wong H.-Y., Chai Y.-Y., Shi C.-W., Amino N., Kikuchi S., Huang S.-H. (2012). Lipid peroxidation dysregulation in ischemic stroke: Plasma 4-HNE as a potential biomarker?. Biochem. Biophys. Res. Commun..

[32] Luo Y., Cui H.-X., Jia A., Jia S.-S., Yuan K. (2018). The Protective Effect of the Total Flavonoids of *Abelmoschus esculentus* L. Flowers on Transient Cerebral Ischemia-Reperfusion Injury Is due to Activation of the Nrf2-ARE Pathway. Oxidative Med. Cell. Longev..

[33] Park D.-J., Shah F.-A., Koh P.-O. (2018). Quercetin attenuates neuronal cells damage in a middle cerebral artery occlusion animal model. J. Vet. Med Sci..

[34] Vrhovsek U., Rigo A., Tonon D., Mattivi F. (2004). Quantitation of polyphenols in different apple varieties. J. Agric. Food Chem..

[35] Keddy P.G., Dunlop K., Warford J., Samson M.L., Jones Q.R., Rupasinghe H.V., Robertson G.S. (2012). Neuroprotective and anti-inflammatory effects of the flavonoid-enriched fraction AF4 in a mouse model of hypoxic-ischemic brain injury. PLoS ONE.

[36] Dong Y.-S., Wang J.-L., Feng D.-Y., Qin H.-Z., Wen H., Yin Z.-M., Gao G.-D., Li C. (2014). Protective effect of quercetin against oxidative stress and brain edema in an experimental rat model of subarachnoid hemorrhage. Int. J. Med. Sci..

[37] Li W.-X., Deng Y.-Y., Li F., Liu B., Liu H.-Y., Shi J.-S., Gong Q.-H. (2015). Icariin, a major constituent of flavonoids from Epimedium brevicornum, protects against cognitive deficits induced by chronic brain hypoperfusion via its anti-amyloidogenic effect in rats. Pharmacol. Biochem. Behav..

[38] Chen S.F., Hsu C.W., Huang W.H., Wang J.Y. (2008). Post-injury baicalein improves histological and functional outcomes and reduces inflammatory cytokines after experimental traumatic brain injury. Br. J. Pharm..

[39] Ruan J., Yao Y. (2020). Behavioral tests in rodent models of stroke. Brain Hemorrhages.

[40] Liu S., Grigoryan M.M., Vasilevko V., Sumbria R.K., Paganini-Hill A., Cribbs D.H., Fisher M.J. (2014). Comparative analysis of H&E and Prussian blue staining in a mouse model of cerebral microbleeds. J. Histochem. Cytochem..

